# Orbital Metastases of Breast Cancer: Case Report and Review of the Literature

**DOI:** 10.32604/or.2026.067876

**Published:** 2026-05-21

**Authors:** Teng Zhu, Siwen Zang, Bo Chen

**Affiliations:** 1 Department of Breast Cancer, Cancer Center, Guangdong Provincial People’s Hospital, Guangdong Academy of Medical Sciences, Southern Medical University, Guangzhou, China; 2 Department of Ophthalmology, Guangdong Eye Institute, Guangdong Provincial People’s Hospital, Guangdong Academy of Medical Sciences, Southern Medical University, Guangzhou, China

**Keywords:** Orbital metastasis, breast cancer, navigation-assisted biopsy, palliative therapy, skeletal metastases, case report

## Abstract

**Background:** Orbital metastases are rare in breast cancer, representing only 3–10% of ocular metastases. This report highlights a case where orbital involvement was the first indicator of systemic metastatic spread. **Case Presentation:** A 72-year-old woman with a history of Estrogen Receptor (ER)-positive (5%), Progesterone Receptor (PR)-negative, Human epidermal growth factor receptor-2 (HER2)-negative breast cancer (diagnosed 3 years prior) presented with right orbital pain, diplopia, and periorbital swelling. Imaging revealed multiple myositis of the extraocular muscles, compressive displacement of the optic nerve, and right periorbital edema. Bone scintigraphy identified multifocal skeletal metastases. A navigation-assisted biopsy confirmed metastatic invasive ductal carcinoma, immunohistochemically consistent with the primary tumor (ER/PR-negative, HER2-negative). A systematic analysis using next-generation sequencing indicated aberrant activation of the phosphoinositide 3 kinase (PI3K)/AKT/mammalian target of rapamycin (mTOR) signaling pathway. Chemotherapy, targeted therapy and bisphosphonate therapy were initiated, with planned radiotherapy for symptomatic progression. **Conclusion:** Orbital symptoms in breast cancer survivors, even subtle ones, necessitate prompt evaluation for metastatic disease. Multimodal imaging (e.g., Computed Tomography (CT)/Magnetic Resonance Imaging (MRI)) combined with image-guided biopsy is critical for diagnosis. Early detection enables multidisciplinary palliative strategies to optimize quality of life while addressing systemic dissemination.

## 1 Introduction

Breast cancer is globally recognized as the most prevalent malignant tumor and a leading cause of cancer-related mortality among women, with a well-documented propensity to metastasize to distant organs such as bone, liver, and lung [1,2,3]. In stark contrast, ocular metastasis represents a relatively rare clinical occurrence, with recent large-scale studies reporting an incidence of approximately 0.5% among all breast cancer patients, which corresponds to roughly 2.4% of those with metastatic disease [4]. This manifestation is frequently underdiagnosed or overlooked in clinical practice, primarily because it often coincides with and is masked by more prevalent systemic metastases, particularly to bone or viscera, leading to a significant diagnostic oversight [4,5]. The anatomical and physiological uniqueness of the orbit substantially contributes to this challenge; its rich vascular supply, especially to the highly perfused choroid, and the immune-privileged microenvironment create a biologically complex niche that is particularly difficult to interrogate with conventional diagnostic modalities [1,5]. Consequently, the true epidemiological burden of breast cancer-related ocular metastasis remains substantially underestimated, resulting in a critical paucity of accurate population-level data and a lack of standardized screening protocols for this specific metastatic manifestation [1]. The reported incidence of ocular metastasis in breast cancer patients varies considerably across different clinical studies, with a broad range from 5% to 30%, a statistic largely influenced by the frequent asymptomatic nature of metastatic foci in the eye compared to other organ systems [6,7]. Notably, orbital metastases are far less frequently encountered than intraocular metastases (which predominantly affect the highly vascular uveal tract, particularly the choroid), accounting for a mere 2% to 3% of all patients with systemic cancer [8,9]. This relative rarity, combined with the fact that orbital metastases are accompanied by metastases in other sites, often leads to their clinical oversight and complicates the accurate determination of their true epidemiological burden [10]. Emerging evidence suggests that the unique orbital microenvironment plays a pivotal role in this context. Characterized by immune privilege, specialized vascular permeability, and dynamic molecular interactions between stromal and tumor cells, this niche may foster metastatic seeds that are resistant to conventional therapeutic strategies [11]. A deeper understanding of these microenvironmental dynamics is crucial, as orbital metastases can be the initial sign of cancer, and they typically present with rapid-onset symptoms such as diplopia, proptosis, and pain, significantly impacting the patient’s quality of life and prognosis [12]. This article presents a rare case of breast cancer with orbital metastasis and discusses the diagnostic approaches, therapeutic interventions, and prognostic outcomes through a comprehensive review of the literature, ultimately aiming to enhance clinical awareness and inform management strategies for this underrecognized metastatic pattern.

The biological mechanisms underlying orbital metastasis remain enigmatic, though recent advances in molecular oncology have begun to unravel potential pathways. Circulating tumor cells that successfully navigate the metastatic cascade may preferentially utilize specific chemokine signaling axes, such as the CXCL12-CXCR4 pathway, to home to and colonize orbital tissues [13]. This homing process is facilitated by the unique vascular architecture of the orbit, particularly the fenestrated capillaries of the highly vascularized choroid and the rich capillary networks within the orbital fat, which provide anchorage points and a fertile soil for disseminated tumor cells to proliferate [14]. Moreover, the immune-privileged status of the orbit, a phenomenon traditionally attributed to mechanisms like blood-tissue barriers, low expression of major histocompatibility complex (MHC) molecules, and high concentrations of immunosuppressive factors, creates a microenvironment characterized by reduced inflammatory responses. This immunosuppressive niche might inadvertently shield metastatic cells from immune surveillance by limiting the infiltration and cytotoxic activity of immune effector cells such as T cells and natural killer (NK) cells, thereby promoting metastatic outgrowth [13,14]. This dual role of the orbital microenvironment—acting as both a physical barrier to systemic therapies and a permissive niche for tumor growth—poses a significant clinical conundrum that demands multidisciplinary solutions.

Clinically, orbital metastases often manifest with nonspecific symptoms, such as unilateral proptosis, diplopia, and periorbital edema, that mimic benign inflammatory conditions, leading to diagnostic delays and therapeutic missteps [15]. The absence of standardized screening protocols for high-risk populations exacerbates this issue, particularly in breast cancer survivors, among whom orbital metastases represent a consequential yet underrecognized late complication [15], allowing metastatic lesions to progress unchecked until irreversible structural damage occurs. A deeper understanding of the clinicopathological correlations between primary breast cancer subtypes and orbital metastatic behavior could refine risk stratification and guide early intervention. Emerging evidence underscores that the cytoskeletal dynamics of cancer cells, regulated by pathways involving PAKs, FAK, and ARF signaling, are pivotal in dictating organ-specific metastatic colonization, including to the orbit [16,17,18]. For instance, hormone receptor-negative tumors, which are inherently more aggressive and prone to visceral spread, may exhibit distinct molecular signatures, such as BRCA mutations or cytoskeletal remodeling proteins like MASTL and Tau, that predispose to orbital colonization [15].

The therapeutic landscape for orbital metastases remains fragmented, with limited consensus on optimal local and systemic strategies. While radiotherapy has long been the cornerstone of local control, emerging modalities such as targeted molecular therapies and immunomodulatory agents offer promise for addressing both the primary tumor biology and the unique challenges of the orbital microenvironment. This case report and literature review seek to bridge these knowledge gaps by synthesizing clinical insights with molecular advancements, ultimately proposing a framework for personalized management of this rare but clinically significant metastatic entity.

This study was approved by the ethics committee of Guangdong Provincial People’s Hospital, with the reference number: KY2024-1183-01. The handwritten informed consent was obtained from the patient. Besides, this study was prepared according to the CARE case report guideline, and a CARE checklist was provided. Please see Supplementary Material S1 for more details [19].

## 2 Case Report

**Figure 1:**
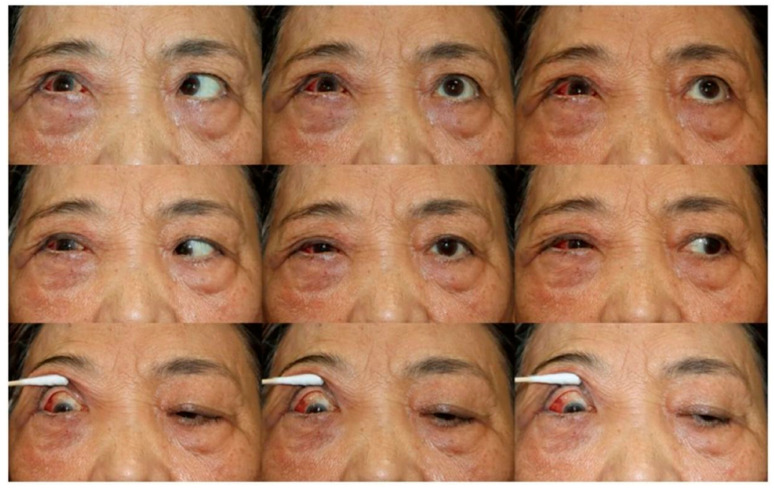
72-year-old female presented with right orbital pain and increased discharge.

A 72-year-old female was admitted to the Cancer Center of Guangdong Provincial People’s Hospital with a three-month history of right and left orbital pain, diplopia, and periorbital swelling (Fig. 1). The patient had been diagnosed with cT2N0 breast cancer three years ago, demonstrating immunohistochemical characteristics of estrogen receptor positivity (ER+ 5%), progesterone receptor negativity (PR-), and human epidermal growth factor receptor 2 negativity (HER2-). Initial therapeutic management included a modified radical mastectomy followed by six cycles of adjuvant chemotherapy with the TC regimen (docetaxel/cyclophosphamide). Then she received endocrine therapy with daily tamoxifen 20 mg after chemotherapy.

**Figure 2:**
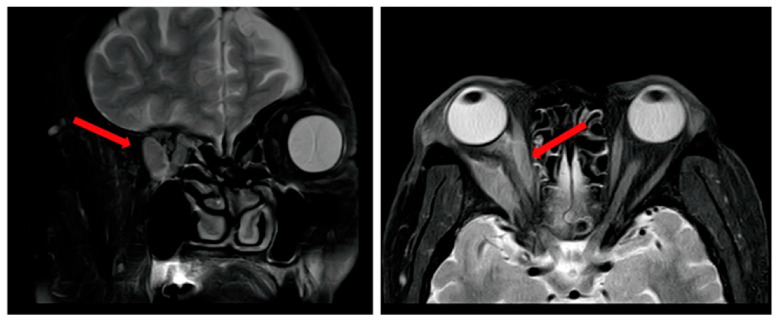
Orbital MRI demonstrated multiple myositis of the extraocular muscles, compressive displacement of the optic nerve, and right periorbital edema. The red arrow represents the compression and displacement of the optic nerve caused by the occupying effect caused by multiple extraocular myositis.

Upon admission, physical examination revealed right conjunctival hyperemia, increased exudate, and visual acuity of 0.7 (corrected to 0.9) in the right eye, compared to 1.0 in the left eye. Orbital Magnetic Resonance Imaging (MRI) demonstrated multiple myositis of the extraocular muscles, compressive displacement of the optic nerve, and right periorbital edema (Fig. 2). Given the clinical suspicion of metastatic malignancy based on medical history, a comprehensive staging evaluation was performed. Subsequent bone scintigraphy and whole-body Computed Tomography (CT) scan revealed extensive bone metastases. Histopathological confirmation through right orbital mass biopsy confirmed the diagnosis of a metastatic invasive ductal carcinoma of the breast with further immunohistochemical profile: ER(-), PR(-), HER2(-). Next-generation sequencing identified an AKT1 exon 4 mutation, indicating aberrant activation of the phosphoinositide 3 kinase (PI3K)/AKT/mammalian target of rapamycin (mTOR) signaling pathway.

**Figure 3:**
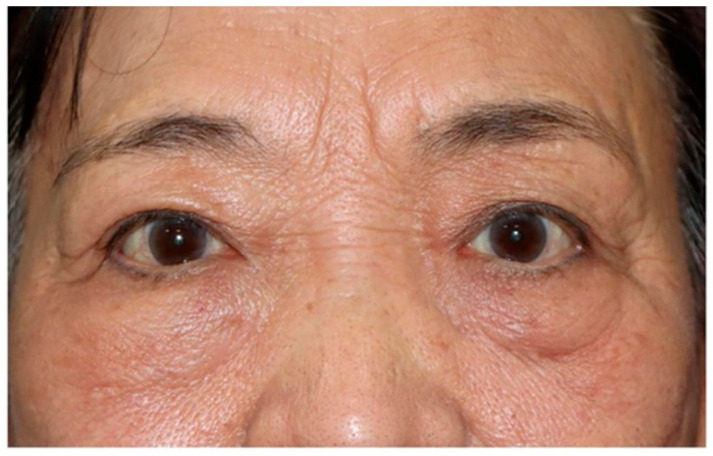
This patient maintained stable systemic disease with significant improvement in ocular symptoms.

**Figure 4:**
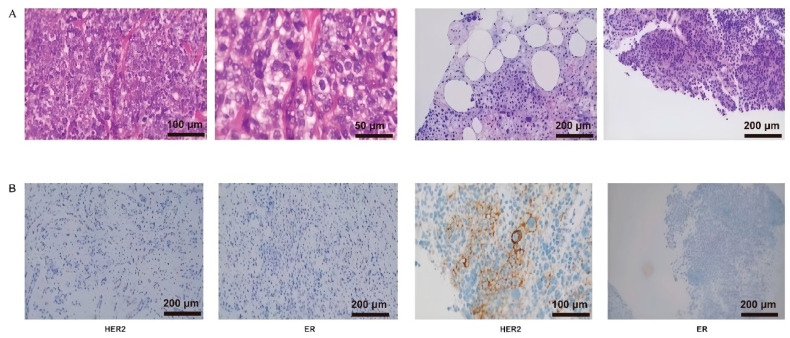
Histopathology of orbital biopsy. (**A**) Hematoxylin and eosin staining showing invasive ductal carcinoma with characteristic glandular structures. (**B**) Immunohistochemical staining was positive for CK7 (left) and GATA3 (right), confirming breast cancer origin.

In accordance with the updated Fudan Classification system for triple-negative breast cancer (TNBC), this case was categorized as Luminal Androgen Receptor (LAR)-PI3K/AKTmut subtype [20]. The therapeutic strategy was informed by next-generation sequencing, revealing an AKT1 exon 4 mutation, prompting the use of everolimus, an mTOR inhibitor, to target downstream signaling in the PI3K/AKT/mTOR pathway. Unlike direct AKT inhibitors (e.g., capivasertib), everolimus inhibits mTORC1, mitigating pathway hyperactivation while avoiding broader toxicity associated with upstream AKT inhibition. First-line therapy with nab-paclitaxel (100 mg/m^2^ days 1, 8, 15) combined with everolimus (10 mg daily) was initiated, supplemented by zoledronic acid (4 mg monthly) for skeletal-related event prevention. During the 6-month follow-up, the patient maintained stable systemic disease with significant improvement in ocular symptoms (Fig. 3). Surveillance MRI showed stable orbital metastases. Subsequent management will incorporate localized radiotherapy for ocular progression if it occurs. Histopathological confirmation through right orbital mass biopsy confirmed metastatic invasive ductal carcinoma of the breast, with immunohistochemical staining positive for Cytokeratin 7 (CK7) and GATA Binding Protein 3 (GATA3), consistent with breast cancer origin (Fig. 4).

**Figure 5:**
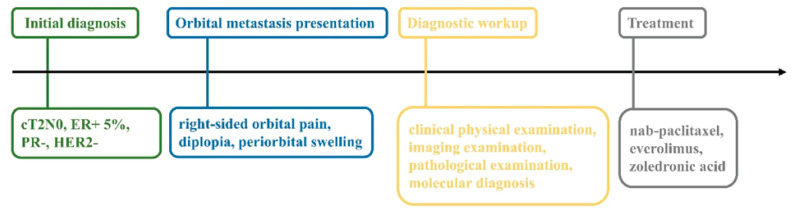
Timeline of clinical events. This figure outlines the sequence from initial breast cancer diagnosis (cT2N0, ER+ 5%, PR-, HER2-) to orbital metastasis presentation (right-sided orbital pain, diplopia, periorbital swelling), diagnostic workup, and treatment initiation with nab-paclitaxel, everolimus, and zoledronic acid.

Additionally, Fig. 5 illustrates the sequence of events from initial breast cancer diagnosis to orbital metastasis presentation, treatment, and follow-up. The timeline clarifies the affected anatomy (right orbit) and key clinical milestones.

## 3 Discussion

Orbital metastases present with a constellation of neuro-ophthalmic symptoms, among which proptosis is observed in 50–75% of cases and ocular motility impairment occurs in up to one-third of patients, primarily due to tumor infiltration of extraocular muscles [11,21]. This infiltration can lead to a restrictive myopathy, manifesting clinically as diplopia and impaired ocular movement. Imaging studies, particularly computed tomography (CT), often reveal diffuse or nodular enlargement of the affected extraocular muscles, which contributes to the mass effect and subsequent proptosis. The pathophysiology involves hematogenous dissemination of tumor cells to the highly vascularized orbital tissues, with subsequent proliferation and microenvironment remodeling driving the symptomatic presentation [22]. Neuropathic orbital pain typically results from perineural invasion, a process often mediated by tumor-neural interactions that can induce peripheral and central sensitization, leading to chronic neuropathic pain states [23], while visual deterioration commonly results from compressive optic neuropathy, which manifests clinically as progressive vision loss, visual field defects, and afferent pupillary defects due to direct mechanical compression and ischemia of the optic nerve. The predilection for orbital involvement may relate to the fenestrated vasculature of the choroid and orbital fat, facilitating tumor cell extravasation via anatomical conduits such as the meningo-orbital foramen, which provides a direct communication between the cranial cavity and the orbit [24]. Recent investigations into tumor-stroma interactions have highlighted the role of extracellular matrix remodeling in orbital metastasis, whereby tumor-derived proteases including matrix metalloproteinases and hyaluronidases degrade local connective tissue to create space for proliferating cells while simultaneously releasing growth factors that stimulate angiogenesis and facilitate further metastatic colonization.

Breast cancer, as the most prevalent primary malignancy leading to orbital metastases, represents approximately 49% of orbital metastases cases, thereby underscoring its significant clinical burden in the field of neuro-ophthalmology [25]. The dissemination predominantly demonstrates hematogenous spread patterns, a process facilitated by the fenestrated vasculature of the choroid and orbital fat which permits efficient tumor cell extravasation and colonization [25]. Notably, clinical observations and emerging data from metastatic mapping studies suggest that orbital involvement typically follows the establishment of pulmonary, hepatic, and central nervous system metastases, implying a sequential pattern of disease progression [26,27]. This observed metastatic hierarchy may be influenced by organ-specific microenvironmental factors, or “soil” determinants, that selectively support the survival and growth of circulating tumor cells arriving at these distant sites [28]. Furthermore, recent investigations into cytoskeletal dynamics have begun to elucidate the mechanistic underpinnings of such organotropism [28], highlighting how molecular adaptations in breast cancer cells, including those regulating cell shape and motility, may predispose them to colonize specific secondary organs like the orbit following initial metastasis to other visceral sites. Accumulating evidence from recent mechanistic and therapeutic studies suggests that chemokine receptors (e.g., CXCR4) and adhesion molecules (e.g., CD44) may mediate organotropism in metastatic breast cancer [29,30]. Specifically, the CXCL12/CXCR4 biological axis is critically involved in forming the pre-metastatic niche (PMN) in distant organs; PMNs persistently release chemokines into the circulatory system, guiding tumor cells to selectively metastasize to specific sites based on chemokine concentration gradients [29]. Innovative strategies targeting this axis, such as using CXCR4-targeted nanothreads to mechanically disrupt receptor dynamics and inhibit downstream signaling, have shown efficacy in suppressing metastatic cascades in breast cancer models [29]. Despite these general advances in understanding organ-specific homing, the orbital-specific homing mechanisms in breast cancer metastasis remain particularly poorly characterized and warrant further dedicated investigation. The metastatic niche within the orbit may further evolve under selective pressures from systemic therapies, fostering resistant subclones with enhanced capacity for immune evasion and metabolic adaptation.

Discordance in estrogen receptor (ER) status between primary and metastatic tumors, as observed in this case (primary: ER+ 5%; metastatic: ER-), is a recognized phenomenon in breast cancer, occurring in 10–30% of cases [31,32]. This receptor conversion, particularly the loss of ER expression in metastases, presents significant challenges for clinical management, as it can directly lead to acquired resistance to endocrine therapies such as tamoxifen or aromatase inhibitors [33]. The underlying mechanisms are multifaceted, involving factors such as tumor heterogeneity, selective pressure from prior endocrine treatments, and epigenetic modifications [31]. For instance, the emergence of ESR1 mutations or activation of alternative survival pathways (e.g., FGFR signaling) under therapeutic pressure can favor the outgrowth of ER-negative tumor clones [34]. Therefore, confirming the receptor status of metastatic lesions through biopsy is crucial for guiding subsequent therapy, as it may necessitate a shift from endocrine-based regimens to chemotherapy or other targeted agents. This underscores that breast cancer is a dynamic disease, and its phenotypic evolution during progression must be considered to optimize patient-specific treatment strategies [34]. This may result from tumor heterogeneity, selective pressure from adjuvant therapies like tamoxifen, or epigenetic silencing of ER expression, potentially contributing to the aggressive phenotype of the metastatic lesion.

Diagnostic challenges persist due to nonspecific early symptoms mimicking benign orbital inflammation or thyroid eye disease. Orbital imaging (MRI with contrast) is critical for distinguishing tumor infiltration from inflammatory pseudotumors, with diffusion-weighted sequences showing >90% sensitivity for malignancy detection [27]. Advanced imaging techniques, including Dynamic Contrast-Enhanced Magnetic Resonance Imaging (DCE-MRI) and diffusion tensor imaging, are increasingly employed to map tumor vascularity and neural infiltration patterns, providing radiologists with multidimensional datasets to differentiate metastatic lesions from inflammatory pathologies. Biopsy remains the gold standard, yet sampling limitations in retrobulbar lesions may necessitate liquid biopsy approaches (e.g., aqueous humor circulating tumor DNA (ctDNA) analysis) in select cases. The integration of liquid biopsies into diagnostic workflows could revolutionize the management of orbital metastases by enabling real-time monitoring of treatment response and early detection of molecular relapse.

The prognosis for patients with orbital metastases remains guarded, reflecting the advanced disease stage and limited efficacy of current therapeutic modalities for metastatic disease in this sanctuary site. Reported survival estimates indicate a median survival of 31 months post-diagnosis and 5-year survival rates below 25% [35,36]. Compared to other metastatic sites, orbital metastases confer a median survival of approximately 35 months, similar to bone metastases (24–36 months) but longer than visceral metastases to the liver or lung (12–24 months) [37]. The coexistence of multiple metastatic sites, as observed in this case, significantly worsens prognosis, emphasizing the need for early detection and aggressive systemic therapy. Multivariate analyses identify HER2-negative status, multiple metastatic sites, and delayed diagnosis as independent predictors of poor outcomes.

The integration of liquid biopsy into oncology has unveiled promising avenues for prognostic refinement. Emerging prognostic biomarkers, such as circulating tumor cells (CTCs) and exosome-derived microRNAs, may refine survival predictions by capturing the dynamic interplay between systemic tumor burden and localized orbital disease progression. CTCs, which are shed from primary or metastatic tumors into the circulation, serve as viable “seeds” for metastasis; their enumeration and molecular characterization (e.g., metabolic profiling) have been shown to correlate with metastatic risk and can provide an early warning of disease progression [38]. Concurrently, exosomes secreted by tumor cells carry a specific molecular cargo, including proteins, DNA fragments, and various RNA species such as microRNAs (miRNAs) and circular RNAs (circRNAs), which can mirror the characteristics of the parent tumor cells and modulate the tumor microenvironment at pre-metastatic niches [39,40]. The combined analysis of these biomarkers offers a synergistic, non-invasive approach to monitor tumor evolution in real-time, potentially overcoming the limitations of traditional imaging and single-timepoint tissue biopsies [38]. For instance, in nasopharyngeal carcinoma, serum exosomal circRNAs have been leveraged to construct prognostic models that effectively stratify patient risk and are associated with distinct immune microenvironment profiles [40]. Despite their potential, challenges remain in standardizing detection protocols and validating clinical utility across diverse cancer types and stages [38]. Future studies focusing on the orbital-specific homing mechanisms and validating these biomarkers in prospective, large-scale cohorts are crucial to translate these promising tools into clinical practice, ultimately enabling more personalized and pre-emptive management strategies for patients with orbital metastases.

Current management protocols emphasize metastasis-directed radiotherapy as the cornerstone of local control for oligometastatic disease, a strategy supported by its integration into multidisciplinary treatment approaches for various cancers [41]. Utilizing precise techniques such as stereotactic body radiotherapy (SBRT) or fractionated stereotactic radiotherapy (FSRT), these regimens deliver high biological effective doses (BED) to the target while minimizing exposure to adjacent organs at risk [42]. For intracranial metastases, common fractionated regimens (e.g., 30–35 Gy in 5 fractions) have demonstrated robust efficacy, achieving tumor volume reduction rates of 63–83% and 1-year local control rates of approximately 87–94% in clinical studies [43]. The high rates of volume reduction are critically important, as a reduction exceeding 65% at 6 months post-treatment has been identified as a significant predictor of superior long-term local control [44]. Furthermore, the optimized dose distribution of modern radiotherapy contributes to its favorable safety profile, with severe (Grade ≥3) toxicity rates typically reported below 6% [41]. Innovative radiotherapy techniques, including intensity-modulated proton therapy, are being explored to minimize collateral damage to sensitive ocular structures while maintaining therapeutic efficacy. Proton beam radiotherapy is emerging as a precision modality, reducing lens dose to <5 Gy compared to 10–15 Gy with photons, potentially mitigating cataract risk in long-term survivors [45]. Surgical intervention is principally reserved for diagnostic biopsy, given the morbidity risks of orbital exenteration.

Notably, 80% of ocular metastases demonstrate disease stabilization during systemic therapy, though metachronous orbital lesions develop in 53% of cases despite treatment [36]. The “orbital sanctuary” hypothesis posits that the blood-ocular barrier may limit drug penetration, necessitating higher drug doses or blood-brain barrier disruptors. This hypothesis gains further credence from preclinical studies demonstrating heterogeneous drug distribution within orbital tissues, with subtherapeutic concentrations observed in retrobulbar adipose compartments compared to vascularized muscle layers [46]. Modern therapeutic paradigms integrate bone-modifying agents (e.g., zoledronic acid 4 mg monthly) with molecularly targeted approaches [47]. The evolving Fudan molecular classification system enables subtype-specific regimens, particularly for PI3K/AKT/mTOR-activated tumors, where everolimus combinations demonstrate median progression-free survival of 13.9 months [48]. The advent of dual-target therapies, which simultaneously inhibit complementary signaling pathways, may overcome resistance mechanisms driven by pathway redundancy and feedback loops.

Quality-of-life considerations warrant emphasis, as 62% of patients experience permanent visual field deficits post-radiotherapy [49]. Multidisciplinary rehabilitation involving low-vision aids and neuro-ophthalmic monitoring is essential. Integrative palliative care models that address both physical symptoms and psychosocial distress—such as chronic pain, disfigurement, and functional disability—are critical for optimizing patient outcomes in the advanced disease setting [49,50,51]. Palliative care integration at diagnosis improves symptom burden scores by 40% in advanced ocular metastasis cohorts [52]. Future directions in orbital metastasis management lie at the intersection of precision medicine and advanced therapeutics. Tumor-agnostic therapies targeting shared molecular vulnerabilities across cancer types, such as DNA damage repair defects or epigenetic dysregulation, may offer new avenues for treatment. Similarly, the development of orbital-specific drug delivery systems—including biodegradable implants and nanoparticle carriers—could enhance therapeutic precision while reducing systemic toxicity.

This case was reviewed at a multidisciplinary team (MDT) meeting, integrating expertise from medical oncology, ophthalmology, radiology, and pathology to formulate a comprehensive management plan. Multidisciplinary care is critical in orbital metastases, as it ensures coordinated systemic therapy, potential radiotherapy, neuro-ophthalmic monitoring, and palliative interventions to optimize both oncologic and quality-of-life outcomes.

This case illustrates the imperative for comprehensive genomic profiling in metastatic TNBC. The observed clinical response (RECIST 1.1 partial response) and 6-month progression-free interval align with phase II trial outcomes of AKT inhibitor combinations [48]. Ongoing surveillance includes quarterly orbital MRI and circulating tumor DNA monitoring to detect emergent drug resistance.

This case report provides a detailed clinicopathological and molecular characterization of a rare orbital metastasis from breast cancer, integrating advanced imaging, histopathology, and next-generation sequencing to guide personalized therapy. The use of navigation-assisted biopsy and comprehensive genomic profiling represents a strength, enabling precise diagnosis and targeted intervention in a clinically challenging site. Multidisciplinary management ensured holistic care, addressing both systemic disease and quality of life. However, several limitations should be acknowledged. As a single-case report, the findings lack generalizability and statistical power. The absence of long-term follow-up limits assessment of sustained treatment efficacy and late toxicities. Furthermore, the receptor discordance between primary and metastatic lesions underscores the dynamic nature of tumor evolution, yet the underlying mechanisms—whether due to sampling bias, clonal selection, or epigenetic modulation—remain speculative without longitudinal molecular monitoring. Future multi-institutional studies with standardized diagnostic and therapeutic protocols are needed to validate our approach and improve outcomes in this rare metastatic setting.

## Data Availability

Not applicable.

## Supplementary Materials

The supplementary material is available online at https://www.techscience.com/doi/10.32604/or.2026.067876/s1.
